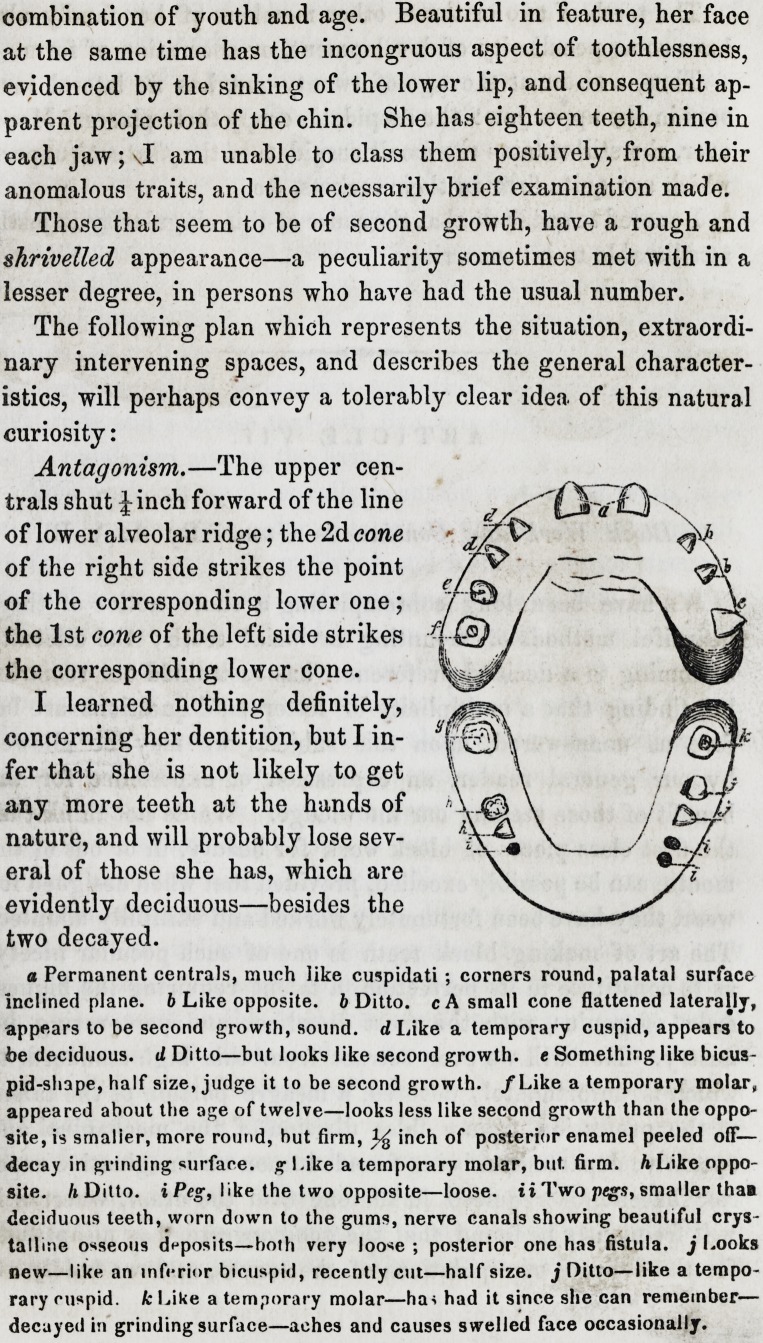# Lusus Naturœ—A Curious Case of Anomalous, and Non-Development of Teeth

**Published:** 1855-01

**Authors:** C. T. Cushman

**Affiliations:** Columbus, Ga.


					ARTICLE VI.
Lusus Naturae?A Curious Case of Anomalous, and Non-de-
velopment of Teeth.
By C. T. Cushman, D. D. S., Colum-
bus, Ga.
A few years ago I examined the mouth of a girl of sixteen,
whose teeth presented an appearance unlike any other I ever
saw. Her temperament is inclined to the strumous, skin exceed-
ingly fair and rose-tinted. Her physiognomy presents a strange
1855.] Case of Anomalous and Non-development of Teeth. 55
combination of youth and age. Beautiful in feature, her face
at the same time has the incongruous aspect of toothlessness,
evidenced by the sinking of the lower lip, and consequent ap-
parent projection of the chin. She has eighteen teeth, nine in
each jaw; \I am unable to class them positively, from their
anomalous traits, and the necessarily brief examination made.
Those that seem to be of second growth, have a rough and
shrivelled appearance?a peculiarity sometimes met with in a
lesser degree, in persons who have had the usual number.
The following plan which represents the situation, extraordi-
nary intervening spaces, and describes the general character-
istics, will perhaps convey a tolerably clear idea of this natural
curiosity:
Antagonism.?The upper cen-
trals shut ^inch forward of the line
of lower alveolar ridge; the 2d cone
of the right side strikes the point
of the corresponding lower one;
the 1st cone of the left side strikes
the corresponding lower cone.
I learned nothing definitely,
concerning her dentition, but I in-
fer that she is not likely to get
any more teeth at the hands of
nature, and will probably lose sev-
eral of those she has, which are
evidently deciduous?besides the
two decayed.
combination of youth and age. Beautiful in feature, her face
at the same time has the incongruous aspect of toothlessness,
evidenced by the sinking of the lower lip, and consequent ap-
parent projection of the chin. She has eighteen teeth, nine in
each jaw; \I am unable to class them positively, from their
anomalous traits, and the necessarily brief examination made.
Those that seem to be of second growth, have a rough and
shrivelled appearance?a peculiarity sometimes met with in a
lesser degree, in persons who have had the usual number.
The following plan which represents the situation, extraordi-
nary intervening spaces, and describes the general character-
istics, will perhaps convey a tolerably clear idea of this natural
curiosity:
Antagonism.?The upper cen-
trals shut J inch forward of the line
of lower alveolar ridge; the 2d cone
of the right side strikes the point
of the corresponding lower one;
the 1st cone of the left side strikes
the corresponding lower cone.
I learned nothing definitely,
concerning her dentition, but I in-
fer that she is not likely to get
any more teeth at the hands of
nature, and will probably lose sev-
eral of those she has, which are
evidently deciduous?besides the
two decayed.
a Permanent centrals, much like cuspidati ; corners round, palatal surface
inclined plane. 6 Like opposite, b Ditto, c A small cone flattened laterally,
appears to be second growth, sound, d Like a temporary cuspid, appears to
be deciduous, d Ditto?but looks like second growth, e Something like bicus-
pid-shape, half size, judge it to be second growth. /Like a temporary molar,
appeared about the age of twelve?looks less like second growth than the oppo-
site, is srnalier, more round, but firm, inch of posterior enamel peeled off-
decay in grinding surface. # Like a temporary molar, but. firm, h Like oppo-
site. h Ditto, i Peg, like the two opposite?loose, ii Two p<g"s, smaller tha?
deciduous teeth, worn down to the gums, nerve canals showing beautiful crys-
talline osseous deposits?both very loose ; posterior one has fistula, j Looks
new?like an inferior bicuspid, recently cut?half size, j Ditto?like a tempo-
rary cuspid, k Like a temporary molar?ha* had it since she can remember?
decayed it) grinding surface?aches and causes swelled face occasionally.
56 Block Worlc and Continuous Gum. [Jan't,
The teeth of two or three other members of her family also
have some peculiarity of development, or aberration of form.
Thus, a sister at the age of twenty-one, has no lateral inci-
sors in the upper jaw; the cuspidati occupy their places. More-
over, she still retains the small cuspidati of the first set, (upper,)
which are quite firm in their sockets, &c.
I am led to conclude that the cause of this singularity is justly
attributable to intermarriage.

				

## Figures and Tables

**Figure f1:**